# A comparison of resting energy prediction equations in young recreationally active women

**DOI:** 10.1186/1550-2783-12-S1-P50

**Published:** 2015-09-21

**Authors:** J Kisiolek, K Schultz, J Luedke, MT Jones, J M Oliver, AR Jagim

**Affiliations:** 1 Exercise & Sport Science Department, University of Wisconsin - La Crosse, La Crosse, WI, 54603, USA; 2 Division of Health and Human Performance, George Mason University, Fairfax, VA, 22030, USA; 3 Kinesiology Department, Texas Christian University, Fort Worth, TX, 76129, USA

## Background

The estimation of resting energy expenditure (REE) can be a valuable tool in developing programs for weight loss interventions and body composition management. REE prediction equations are a low-cost alternative to assess REE versus directly measuring REE, which typically requires expensive laboratory equipment. Though often used to estimate REE in active populations, the majority of REE equations have been developed in overweight or sedentary populations. This study sought to examine the accuracy of three commonly used REE estimation equations in a recreationally active population.

## Methods

Twenty-five recreationally active, college-aged women (20.72 ± 0.97 yrs; 163.04 ± 5.67 cm; 67.08 ± 10.40 kg; 29.04 ± 5.80% BF) were recruited to participate in this observational study. Participants underwent a single day of testing, consisting of determination of REE by indirect calorimetry (*TrueOne^® ^2400 Metabolic Measurement system, ParvoMedics, Sandy, UT*) followed by body composition assessment. Participants were instructed to refrain from strenuous exercise 48 hrs prior to testing in addition to fasting >8 hrs prior. Participants laid motionless without falling asleep for 15-20 minutes during REE determination. Data were recorded during a period of time in which criterion variables (e.g., VO_2 _L/min) changed less than 5% every 5 minutes. Body composition was assessed using air displacement plethysmography (*BODPOD, Cosmed, USA*). Fat and fat-free mass were determined based upon the body densities obtained from the BODPOD and the Siri equation. Independent sample t-test was used to determine the difference between indirect calorimetry and each of the following REE prediction equations: 1) Nelson Equation; 2) Mifflin-St. Jeor Equation; and 3) Harris-Benedict Equation (with a moderate activity factor). Bivariate Pearson correlations were also used to determine the relationship between methods of REE assessment. A criterion alpha level of p < 0.05 was selected to determine statistical significance.

## Results

All three REE equations were significantly different than indirect calorimetry (p < 0.001; Table 1). The Nelson and Mifflin-St. Jeor equations underestimated REE when compared to indirect calorimetry by 345.5 ± 51.5 and 220.6 ± 47.3 kcals, respectively; while the Harris Benedict overestimated REE by 272.4 ± 49.3 kcals. All three equations were moderately correlated with REE as determined by indirect calorimetry.

**Table 1:** Comparison of REE prediction equations.

	REE (kcal) Mean ± SD	t-test p value	r value	p value
**Indirect Calorimetry**	1646 ± 204.6			

**Nelson Equation**	1301.3 ± 155.9	p < 0.001	0.687	p < 0.001

**Mifflin-St. Jeor Equation**	1426.2 ± 118.5	p < 0.001	0.630	p < 0.001

**Harris-Benedict Equation**	1919.2 ± 137.2	p < 0.001	0.682	p < 0.001

Values are × ± SD; r represents Pearson correlations; P values represent 2-tailed testing.

## Conclusions

Results of the current study suggest that REE prediction equations differ from directly assessed REE using indirect calorimetry. Practitioners should exercise caution when providing dietary recommendations based upon predicted REE values as certain equations may over or underestimate energy requirements by several hundred kilocalories.

